# Binding of a single nitric oxide molecule is sufficient to disrupt DNA binding of the nitrosative stress regulator NsrR†

**DOI:** 10.1039/d4sc04618h

**Published:** 2024-10-15

**Authors:** Jason C. Crack, Nick E. Le Brun

**Affiliations:** a Centre for Molecular and Structural Biochemistry, School of Chemistry, Pharmacy and Pharmacology, University of East Anglia Norwich Research Park Norwich NR4 7TJ UK j.crack@uea.ac.uk n.le-brun@uea.ac.uk

## Abstract

The regulatory protein NsrR, a member of the Rrf2 protein superfamily, plays a major role in the cellular response to nitrosative stress in many benign and pathogenic bacteria. The homodimeric protein binds a [4Fe–4S] cluster in each subunit (termed holo NsrR), and represses transcription of genes primarily involved in NO detoxification. Holo NsrR reacts rapidly with multiple NO molecules per [4Fe–4S] cluster, *via* a complex reaction, with loss of DNA binding and formation of NsrR-bound iron-nitrosyl species. However, the point at which DNA binding is lost is unknown. Here, we demonstrate using surface plasmon resonance (SPR) and native mass spectrometry (MS) that holo NsrR binds the promoter regions of NsrR-regulated genes with promoter-dependent nanomolar affinity, while hemi-apo NsrR (*i.e.* one cluster per dimer) binds >10-fold less tightly, and the cluster-free (apo) form not at all. Strikingly, native MS provided detailed information about the reaction of NO with the physiologically relevant form of NsrR, *i.e.* DNA-bound dimeric NsrR. Reaction with a single NO molecule per NsrR dimer is sufficient to abolish DNA binding. This exquisite sensitivity of DNA binding to NO is consistent with the importance of de-repressing NO detoxification systems at the earliest opportunity to minimise damage due to nitrosative stress. Furthermore, the data show that previously characterised iron-nitrosyls, which form at higher ratios of NO to [4Fe–4S], are not physiologically relevant for regulating the NsrR on/off switch.

## Abstract 2

The mechanism of NO sensing by the nitrosative stress regulator NsrR is elucidated. Binding of a single NO to one of the [4Fe–4S] clusters of dimeric NsrR results in dissociation of its complex with DNA.

## Graphical abstract

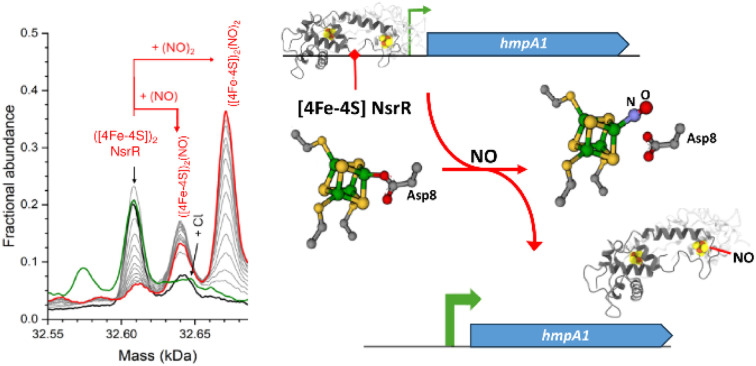

## Introduction

Nitric oxide (NO) is a ubiquitous signalling molecule and cytotoxic agent that has a well-documented role in both prokaryote and eukaryote biology.^1–3^ As a free radical, NO seeks to stabilise its unpaired electron by spin pairing with other species containing unpaired electrons, or through an interaction with the d-orbitals of transition metals.^4^ Hence, NO readily reacts with O_2_ and other radicals, *e.g.* superoxide (O_2_^−^), to generate reactive nitrogen species (RNS), including peroxynitrite (ONOO^−^) and, where thiols are also present, *S*-nitrosothiols (RS-NO).^4^ The interaction of NO with transition metals, principally iron, results in a diversity of iron-nitrosyl species that may be protein-bound (principally, R–Fe(NO) and R–Fe(NO)_2_) in biological samples following the interaction of NO with iron-sulfur cluster-containing proteins.^5–7^

Inhibition of growth lies at the centre of bacterial NO physiology, being derived from either exogenous (*e.g.* host immune response towards pathogens, or bacterial denitrification) or endogenous NO sources. For bacteria, such as *E. coli*, endogenous NO production is mainly catalysed during O_2_ limitation by reaction of nitrite (NO_2_^−^) with cytoplasmic nitrate reductases.^8–11^ This may also be the case for *Streptomyces* sp., some of which can reduce nitrate (NO_3_^−^) to NO_2_^−^.^12^ Though unable to support growth, nitrate respiration allows spores and mycelia to remain metabolically active, enhancing survivability of these obligate aerobes.

The model organism *Streptomyces coelicolor* A3 contains three non-redundant respiratory nitrate reductases, termed Nar1–3, with each Nar complex making a distinct contribution to energy conservation during different stages of the *Streptomyces* life cycle;^13–16^ Nar1 is exclusive to spores, Nar2 to growing mycelium and Nar3 to stationary phase mycelium.

The inherent reactivity of Fe–S clusters towards NO, while potentially deleterious, has resulted in the evolution of proteins, such as NsrR, that function as sensor-regulators.^17–19^ NsrR belongs to the Rrf2 protein superfamily and is found in a wide range of benign and pathogenic bacterial species,^20–24^ and is particularly common in members of the Enterobacteriaceae genera of gamma-proteobacteria, as well as in some Gram-positive bacteria belonging to the Actinobacteria (primarily *Corynebacteriale* and *Streptomycetale*) and *Firmicutes* (mainly *Bacilli*). Thus, NsrR appears to be advantageous to commensal, pathogenic and saprophytic species alike, suggesting nitrosative stress is a common factor of microbial life.^11^

NsrR binds a [4Fe–4S] cluster and, where studied, functions to repress transcription of regulated genes in the absence of NO.^22,25^ Transcriptomic analysis of multiple NsrR regulons has revealed a suite of genes primarily involved in NO detoxification. The principal target in most species is the *hmp* gene, which encodes a flavohemoglobin that converts cytoplasmic NO to NO_3_^−^ under (micro)aerobic conditions.^22,26–29^ In *S. coelicolor* A3 HmpA1 (SCO7428) and NsrR (SCO7427) collaborate to maintain NO homeostasis and attenuate the ability of the DevSR two-component system to sense and respond to NO *in vivo*.^30^ We note that NO plays a role in the physiology of *Streptomyces coelicolor* A3 colonies, modulating secondary metabolite production (*e.g.* production of blue-pigmented antibiotic actinorhodin), and even aerial mycelium formation.^3,30,31^

The NsrR isolated from *S. coelicolor* A3 is the best characterised to date, with reported crystal structures of apo and holo forms and the holo NsrR-DNA complex (see PDB: 5N08, 5N07 and 7B0C, respectively).^32–34^ NsrR is dimeric and features an elongated fold typical of Rrf2 transcriptional regulators.^32,34,35^ Each monomer is composed of eight α-helices and two anti-parallel β-strands arranged to form a winged helix-turn-helix (wHTH) DNA-binding domain and dimerization helix, in addition to a cluster-binding loop. The latter provides three ligands (Cys93, 99 and 106) to the [4Fe–4S] cluster, resulting in well-defined loop structure (Fig. S1†).

**Fig. 1:**
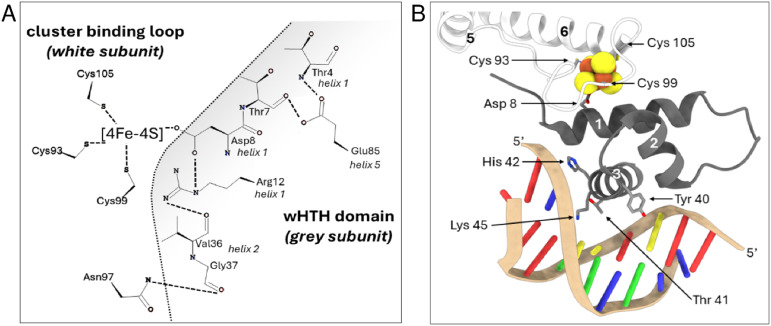
Close up view of the [4Fe–4S] NsrR-*hmpA1* promoter interface. (A) 2D representation of interaction networks near the [4Fe–4S] cluster. Arg12 from helix 1 connects to Asp8 from the same helix and Val36 from helix 2 of the wHTH domain. Glu85 connects helix 5 to Thr4 and Thr7 of helix 1. Gly37 from helix 2 forms an inter-subunit connection with Asn97 in the cluster-binding loop of the other subunit. These interactions, together with the [4Fe–4S] cluster, correctly position the recognition helix of each subunit for optimal DNA binding (PDB: 7B0C). (B) 3D arrangement of cluster binding loop (white subunit) and wHTH domain from the adjacent subunit (grey subunit) when bound to DNA. The white monomer provides three ligands (Cys 93, 99, 105) to the [4Fe–4S] cluster (in space filling representation) resulting in a well-defined loop structure around the cluster. The fourth ligand, Asp 8, is located close by on the ancillary helix (helix 1) of the wHTH domain from opposite monomer (grey subunit), helping to optimally position the recognition helix (helix 3, grey subunit) for DNA binding. Residues of the recognition helix that ‘read’ the nucleotide sequence are also shown.

The fourth cluster ligand, Asp8, is located close by on the ancillary helix of the wHTH domain of the opposite monomer; a characteristic unique to Fe–S Rrf2 transcriptional regulators. Asp8 forms a salt bridge with Arg12 from the same helix, which also interacts with the carbonyl oxygen atom of Val36, located within the wHTH domain. An inter-subunit H-bond between Gly37 and Asn97 provides an additional contact between the wHTH domain and the cluster-binding loop. Glu85 provides additional contacts to the ancillary helix through H-bonds with the main chain N of Thr4 and the O of Thr7. Together, these interactions optimally position the recognition helix in a cluster-dependent manner ready for DNA binding^32,34,36^ (Fig. 1A).

When bound to DNA (*hmpA1* promoter) NsrR makes numerous contacts with the nucleic acid phosphate backbone, and few specific interactions between amino acid side chains and nucleobases. Only Tyr40, Thr41, His42 and Lys45 from the recognition helix, and Arg60 from the wing of the wHTH domain interact directly with the nucleobases^33^ (Fig. 1B). In the absence of the cluster, the Arg12-Val36, Gly37-Asn97 and Glu85-Thr4/Thr7 interactions are disrupted, resulting in a repositioning of the recognition helix and loss of DNA binding. Other notable changes include the C-terminal lengthening of helix 5 to include Cys93, resulting in an ‘opening’ of the cluster-binding loop. Aps8 or Glu85 to Ala substitutions are sufficient to decouple the presence/state of [4Fe–4S] cluster from DNA binding.^34^ This suggests relatively minor modifications of the cluster environment are sufficient to abolish DNA binding.^34,36^

Using a range of spectroscopic, kinetic, and particularly mass spectrometric approaches, we have previously shown that [4Fe–4S] NsrR reacts rapidly in a complex reaction involving multiple NO molecules per cluster, resulting in forms of NsrR with bound iron-nitrosyl species that are closely related to well-characterised small molecule iron-nitrosyls such as dinitrosyl iron complex (DNIC), Roussin's red ester (RRE) and Roussin's black salt (RBS).^6,7,37–41^ Intriguingly, electrophoretic mobility shift assays (EMSAs) indicated that the ratio of NO to cluster required to abolish DNA binding was different for the three known *S. coelicolor* NsrR-regulated promoters, *nsrR* (SCO7427), *hmpA1* (SCO7428), *hmpA2* (SCO7094), suggestive of a hierarchical response to NO, *via* an unknown mechanism.^37^

Here we aimed to gain a much better understanding of the NsrR species interacting with DNA than is available from EMSAs alone.^42^ By applying surface plasmon resonance (SPR) and native mass spectrometric methods to detect and quantitate DNA binding by NsrR and the reaction of DNA-bound NsrR with NO, we establish a new model for how NsrR functions as a sensor-regulator of nitrosative stress.

## Materials and methods

### Purification of *S. coelicolor* NsrR

NsrR was over-produced in aerobically grown *E. coli* (BL21 λDE3) cultures harbouring pNsrR (non-tagged NsrR) or pJM002(C-terminal His-tagged NsrR), as previously described.^22,43^ NsrR was purified anaerobically and, where necessary, the [4Fe–4S] content was enhanced, *in vitro*, *via* a standard NifS-catalysed reconstitution, as previously described.^22,43^ Protein concentrations were determined using the methods of Smith *et al.* (Pierce)^44^ with bovine serum albumin as the standard. Cluster content was determined using an extinction coefficient of *ε*_406nm_ = 13.30 (±0.19) mM^−1^ cm^−1^.^22^

### Surface plasmon resonance (SPR)

Single stranded oligonucleotides, designed around the ReDCaT principle, were purchased from Eurofins Genomics.^45^ ReDCaT oligos were diluted to 100 μM in DNAse-free water and annealed by heating (as previously described^46^), to give 50 μM dsDNA. NsrR was initially exchanged into 0.1 M HEPES, 1.5 M NaCl, 0.5% (v/v) polysorbate 20, pH 7.4 *via* Zeba spin desalting columns (∼7 kDa MWCO, Thermo Scientific), to give a stock solution of ≥60 μM [4Fe–4S] NsrR. All SPR measurements were performed at 25 °C on a Bioacore S200 (Cytiva) using a multi cycle kinetics protocol.^47^ Biotinylated dsDNA ReDCaT was diluted to ∼1 nM with SPR buffer (10 mM HEPES, 150 mM NaCl, 0.05% polysorbate 20, pH 7.4) and captured on streptavidin sensor chips (Xantec) to a density of ∼30 response units (RU). Next, complementary ReDCaT oligos were dissociated from the working surfaces using regeneration buffer (1 M NaCl, 50 mM NaOH), and the complementary strand replaced with a complementary ReDCaT sequence containing a recognised dSDNA NsrR-binding site (Table S1†). Loosely attached material was removed from the chip surface by two 60 μL injections of 2 M NaCl in SPR buffer. NsrR was diluted in SPR buffer to the required concentrations (0–32 nM) and injected in parallel over the immobilised dsDNA surfaces. The association phase was 180 s or 240 s for *hmpA1* and *hmpA2*, respectively, followed by a 750 s dissociation phase. The flow rate was 50 μL min^−1^ throughout. Between runs, chip surfaces were washed with 2 M NaCl in SPR buffer to disrupt protein-DNA interactions.^47^ Sensorgrams were recorded with a 40 Hz data rate from each flow channel (Fc1 to 4) and referenced against a channel Fc1 containing the immobilised ReDCaT probe. Experiments were performed in triplicate for each protein concentration, and two independent runs were performed. Immobilised DNA surfaces were refreshed daily using the appropriate complementary strand.

### Processing of SPR sensorgrams

Reference subtraction and baseline corrections were performed automatically by Biacore S200 evaluation software (Cytiva). Data sets were then exported and initially analysed by Anabel^48^ to provide estimates of the association (*k*_a_) and dissociation (*k*_d_) rate constants. These were used as the starting parameters for kinetic fitting in the Biacore S200 evaluation software. For global fitting, the association and dissociation phases were simultaneously fitted for five different [4Fe–4S] NsrR concentrations. A bivalent model was used because fitting to a typical monovalent analyte-ligand kinetic binding model (Langmuirian) was not satisfactory.

### Preparation of oligonucleotides for ESI-MS

High purity, salt-free, self-complementary, single stranded oligonucleotides were purchased from Eurofins (Eurofins Genomics) and dissolved in DNAse-free water to give 200 μM stock solutions. Double stranded DNA oligonucleotides (dsDNA), containing NsrR promoter sequences, were annealed from equimolar concentrations of single stranded oligonucleotides following heating to 70 °C for 10 min. After cooling, dsDNA was exchanged into 100 mM ammonium acetate, pH 8 *via* Zeba spin desalting columns (∼7 k Da MWCO, Thermo Scientific). The dsDNA content was determined using the sum of the extinction coefficients for the appropriate single stranded oligonucleotides (Table S2†).

### ESI-MS under native conditions

Samples of NsrR were initially exchanged into 1 M ammonium acetate, pH 8.0 using PD Minitrap, G-25 columns (Cytiva) to give stock solutions of ≥100 μM cluster, that could be stored anaerobically at −35 °C for prolonged periods. Prior to ESI-MS analysis aliquots of the stock solution were diluted to 8 μM cluster with 100 mM ammonium acetate pH 8 (200 μL final volume). To study the effect of DNA, increasing aliquots of promoter DNA were added to the diluted sample. Samples were transferred from the anaerobic cabinet using a gas tight syringe and infused directly (5 μL min^−1^) into the source of microTOF-QIII mass spectrometer operating in the positive ion mode. The ESI-TOF was calibrated using ESI-L low concentration tuning mix (Agilent Technologies). Mass spectra (*m*/*z* 1000–6000) were recorded for 5 min with acquisition controlled by Bruker oTOF control software, with parameters as follows: dry gas flow 4 L min^−1^, nebulizer gas pressure 0.8 bar, dry gas 180 °C, capillary voltage 3.5 kV, offset 0.5 kV, quadrupole ion voltage 5 V, collision RF 3.5 kVpp, collision cell voltage 10 V. Processing and analysis of MS experimental data were carried out using Compass Data Analysis version 4.1, with Maximum Entropy v1.3 (Bruker Daltonics, Coventry). Prior to global analysis, MS intensity data was processed to generate fractional abundance plots of ions counts for the relative species as a fraction of the total ion count for all species, as previously described.^38,49,50^ Global analysis was then performed using Dynafit 4 (BioKin, Ltd).^51^ Some variations were observed, which are represented by error bars in the fractional abundance plots.

### Nitrosylation of samples during native MS

It was previously shown that the nitrosylation of NsrR is extremely rapid and that a slow NO-releasing reagent can be used for native *in situ* nitrosylation ESI mass spectroscopy experiments.^38^ Under these conditions, NO availability limits the reaction, enabling an effective thermodynamic titration of the samples. An aliquot (5 μL) of working DEA NONOate solution was added to the NsrR-DNA complex (200 μL, containing 8 μM [4Fe–4S] (4 μM dimer), 8 μM dsDNA) in 100 mM ammonium acetate. The sample was loaded into a gas-tight syringe, maintained at a constant temperature (30 °C), and infused directly into the ESI source, operating in positive ion mode. Spectra were continuously recorded and averaged every 2 min. Stock solutions of DEA NONOate (∼60 mM) were prepared in a native MS compatible alkaline carrier solution (250 mM ammonium acetate pH 8, 1 M ammonium hydroxide), quantitated by absorbance, *ε*_250nm_ = 6.5 mM^−1^ cm^−1^, (Cayman Chemicals, USA), and frozen until needed. Working DEA NONOate solutions were prepared immediately before use by diluting an aliquot of the stock into pre-chilled (∼4 °C) 100 mM ammonium acetate pH 8 using a Starchill MCT microcentrifuge rack (Starlab) to give a 600 μM solution. DEA NONOate spontaneously dissociates in a pH-dependent manner by a first-order process, yielding 1.5 NO molecules per NONOate. In ammonium acetate pH 8, DEA NONOate decayed with a half-life (*t*_½_) of 17 or 8 min at 25 or 30 °C, respectively.^52^ For fitting of thermodynamic titrations, fractional abundances were calculated for specific species present during titrations from total ion counts and fitted using the program Dynafit (Biokin)^51^ according to the reaction scheme comprising eqn (1) and (2), where P is protein and D is DNA.

1 PD + NO ↔ P(NO) + D

2 P + NO ↔ P(NO)

Eqn (1) represents the coupled binding of NO to NsrR-DNA and the subsequent dissociation of nitrosylated NsrR from DNA, and therefore does not correspond to a simple (single) binding process. However, because the affinity of nitrosylated NsrR for DNA is low, it is assumed that the reaction is controlled by the NO-binding step, *i.e.* the affinity of NsrR-DNA for NO. Eqn (2) represents only the NO-binding step, and simultaneous fitting of data for NO binding to DNA-bound and non-DNA-bound NsrR to yield a single *K*_d_ would only be possible if the above assumption is correct.

## Results

### Binding of [4Fe–4S] NsrR to DNA detected by native mass spectrometry

Previous native mass spectrometric studies of His-tagged [4Fe–4S] NsrR in the absence of DNA gave an *m*/*z* spectrum comprising two regions, corresponding to monomeric and dimeric forms of the protein.^38,53^ Here, we generated a non-tagged form of [4Fe–4S] NsrR and found that it preferentially ionised as a dimer, yielding higher intensity signals with better resolved peaks (Fig. S2†). Hence, the non-tagged form of [4Fe–4S] NsrR was used for all subsequent experiments.

**Fig. 2:**
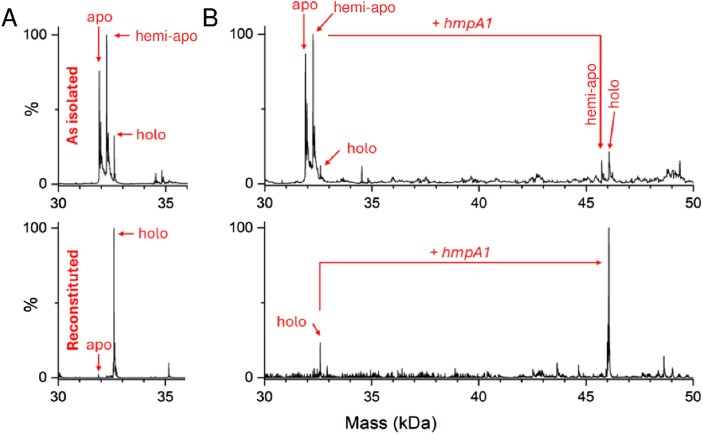
Native MS [4Fe–4S] NsrR and NsrR-*hmpA1* complexes. (A) Deconvoluted spectra of as-isolated and reconstituted [4Fe–4S] NsrR samples (8 μM [4e–4S]), as indicated. As-isolated NsrR is heterogeneous, containing apo, hemi-apo and holo NsrR dimers. Reconstituted NsrR is more homogeneous, containing a high proportion of holo NsrR dimers. (B) Deconvoluted spectra of equivalent samples (8 μM [4e–4S]) treated with *hmpA1* DNA (8 μM) resulting in the appearance of [4Fe–4S] NsrR-DNA complexes. Samples were ionised from 100 mM ammonium acetate, pH 8.0 in positive mode. See Table S2 and Fig. S3† for further details.

As isolated, this non-tagged NsrR samples were ∼60% cluster loaded. The deconvoluted mass spectrum (Fig. 2A and S3A†) of non-tagged [4Fe–4S] NsrR revealed three distinct peaks at 31 907, 32 257 and 32 608 Da, corresponding to dimeric apo NsrR (31 907 Da, ∼75% relative intensity), and dimeric NsrR containing one (32 257 Da, ∼100%) or two clusters (32 608 Da, ∼32%), respectively (see Table S3† for a comparison between observed and predicted masses). This showed that as-isolated samples are clearly heterogeneous. Thus, an *in vitro* cluster reconstitution was performed to generate a more homogeneous sample of [4Fe–4S] NsrR. The deconvoluted MS spectrum of reconstituted NsrR was dominated by dimeric NsrR containing two clusters (32 608 Da, ∼100% relative intensity, hereafter referred to as holo NsrR), with dimeric apo NsrR (hereafter referred to as apo NsrR) and NsrR containing one cluster (hereafter referred to as hemi-apo NsrR) accounting for the remaining ∼3% (Fig. 2A and S3A†).

The addition of 22 bp dsDNA (16 μM) containing the *hmpA1* recognition sequence (13 466 Da) to either as-isolated, or reconstituted NsrR samples caused new charge states to appear at ≥3000 *m*/*z*. These new charge states, upon deconvolution, were found to correspond to DNA complexes of NsrR. DNA complexes involving holo NsrR (46 073 Da) were common to both sample types, but DNA complexes of hemi-apo NsrR (45 723 Da) were unique to the heterogeneous, as-isolated sample (Fig. 2B and S3B†).

### Binding of [4Fe–4S] NsrR to DNA detected by surface plasmon resonance

Surface plasmon resonance (SPR) enables high-sensitivity measurements of the binding of analyte species (NsrR) to an immobilised ligand (DNA) and, depending on the degree to which the chip surface is modified, may provide association (*k*_a_) and dissociation (*k*_d_) rate constants and the equilibrium dissociation constant (*K*_d_).^45–47,54^ To begin with, we investigated the binding of holo NsrR to the high affinity *hmpA1* promoter by SPR.

**Fig. 3:**
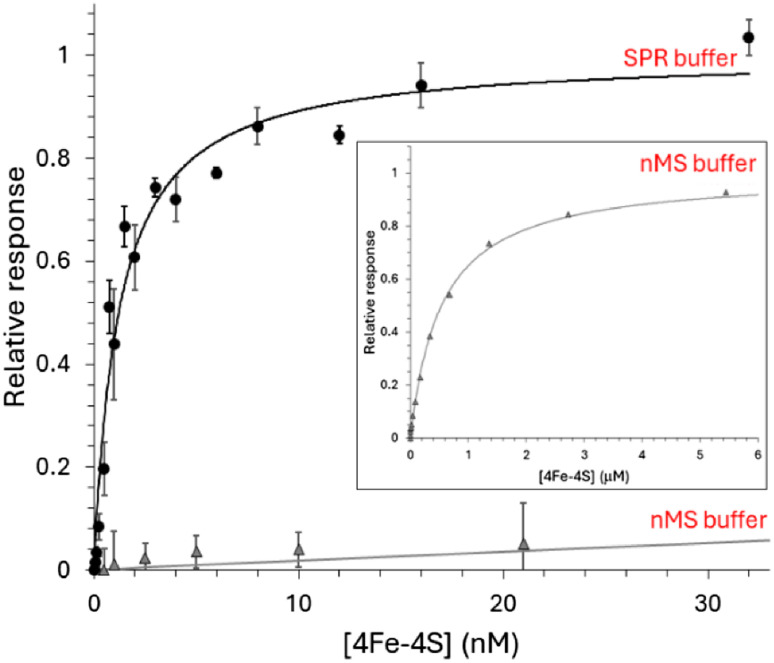
Formation of [4Fe–4S] NsrR-*hmpA1* complexes probed by SPR. Analyte binding response of reconstituted [4Fe–4S] NsrR to 29bp *hmpA1* promoter region in SPR buffer (black circles) and modified native MS buffer (grey triangles). The data were fitted using a simple binding equation, giving a *K*_d_ of 1.2 (±0.2) nM (*n* = 12) for [4Fe–4S] NsrR in SPR buffer (black line). Inset shows the effect of modified native MS buffer on DNA binding, which was weaker, with a *K*_d_ of 0.54 (±0.1) μM (grey line). SPR was performed at 25 °C with SPR buffer (10 mM HEPES, 150 mM NaCl, 0.05% polysorbate 20, pH 7.4) and modified native MS buffer (100 mM ammonium acetate, 0.05% polysorbate 20, pH 8.0).

Initially, we utilised the ReDCaT principle of SPR to obtain the binding affinity of NsrR for *hmpA1*.^45,46^ Binding was found to be specific, such that negligible binding of NsrR to the reference surface containing just the immobilised ReDCaT probe was detected, and satisfactory fits to the data could be obtained using a simple binding equation, giving a *K*_d_ of 1.2 nM, Fig. 3. These data were qualitatively similar to previous EMSA observations.^22^ We then repeated the SPR measurements using a modified version of the volatile ammonium acetate buffer necessary for ESI-MS measurements (100 mM ammonium acetate, 0.05% polysorbate 20, pH 8.0). Again, a satisfactory fit of the data (Fig. 3, inset) was obtained using a simple binding equation, but with a *K*_d_ of 0.54 μM, indicating that DNA binding is much weaker in the presence of ammonium acetate, likely due to competitive binding. We note that holo NsrR makes numerous contacts with the negatively charged phosphate backbone of *hmpA1* (PDB: 7B0C) and that acetate salts are commonly used in the purification of DNA to reduce contamination from DNA binding proteins.^33,55^

### Different NsrR species have different affinities for the *hmpA1* promoter

The ability of native MS to resolve the different NsrR species and the complexes they form with DNA has potential to provide new insight not available from EMSA or SPR experiments. For native MS, *in vitro* reconstituted samples containing 8 μM [4Fe–4S] NsrR (∼4 μM dimer) in 100 mM ammonium acetate, pH 8 were optimal for resolution and signal to noise. Lower concentrations were evaluated, but these typically required extended acquisition times to achieve the same quality of signal, while sub-micromolar concentrations were not useful.

**Fig. 4:**
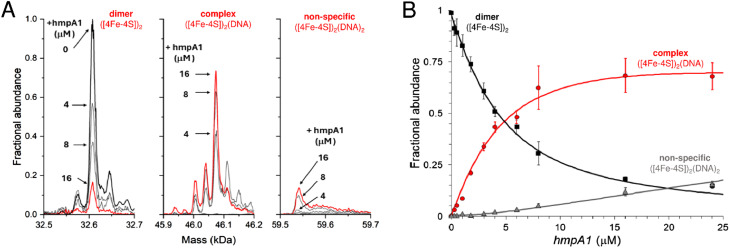
Formation of [4Fe–4S] NsrR-*hmpA1* complexes probed by native MS. (A) Deconvoluted mass spectra at selected concentrations of *hmpA1* DNA showing the formation of NsrR-*hmpA1* complexes from NsrR dimers (8 μM [4Fe–4S]), as indicated. At higher concentrations of *hmpA1* non-specific NsrR-*hmpA1* complexes appear, involving two DNA molecules. See Fig. S3† for annotation of adducts. (B) Plots of fractional abundance for dimeric, complexed, and non-specific NsrR species as a function of the *hmpA1* concentration. Solid lines show fits of the data to a simple sequential binding model, giving a *K*_d_ of 3 (±0.1) μM for NsrR-*hmpA1* complexes and a *K*_d_ of 170 (±70) μM for the non-specific complex. Proteins and complexes were ionised from 100 mM ammonium acetate, pH 8, with positive mode ESI.†

To determine the affinity of holo NsrR for DNA using native MS, 8 μM [4Fe–4S] (∼4 μM dimer) was titrated with increasing concentrations of *hmpA1* promoter DNA. This caused a gradual progression from unbound holo NsrR to DNA-complexed holo NsrR. At higher concentrations of *hmpA1*, holo NsrR in complex with multiple DNA molecules was also observed (Fig. 4A). The fractional abundances of unbound and DNA-complexed NsrR species were analysed according to a simple equilibrium binding model. The resulting fit of the data revealed that holo NsrR bound to *hmpA1* with an affinity of *K*_d_ = ∼3 μM, similar to, but somewhat weaker than, that from SPR measurements. The interaction of holo NsrR with multiple *hmpA1* molecules had a *K*_d_ of ∼170 μM, suggesting this may be the origin of the non-specific DNA binding observed by EMSA (Fig. 4B).^34^ Thus, in comparison to SPR measurements with standard SPR buffers, the affinity of holo NsrR for *hmpA1* determined by native MS was significantly lower, but generally consistent with SPR measurements employing a buffer similar to that used for native MS. There are many examples in which the behaviour of proteins, or protein complexes, in the gas phase closely mimics that in solution. However, here there is a clear effect on the affinity of holo NsrR for DNA measured by native MS due to the requirement for volatile buffers and/or its transition to the gas phase that result in competition. Therefore, the affinities derived from native MS experiments should be considered as relative affinities, rather than absolute.

Next, we sought to establish if holo NsrR, despite the reduced affinity, could discriminate between cognate DNA, *e.g. hmpA1*, and a related, non-cognate promoter. To achieve this, DNA carrying the promotor region from *Corynebacterium glutamicum hmp* (*Cg hmp*) was used in place of *hmpA1*. The *Cg hmp* promoter is comparable in sequence to *hmpA1* (Fig. S4A†) but lacks many of the specific bases that have been identified as directly interacting with amino acid residues from the NsrR wHTH domain.^33^ We also note that *Cg hmp* is not regulated by NsrR and that no proteins of significant similarity to NsrR are found in *C. glutamicum*.^56^ Following the addition of 16 μM *Cg hmpA* to holo NsrR (8 μM [4Fe–4S], ∼4 μM dimer), some holo NsrR-DNA complex was present (∼40% relative intensity), with the majority being unbound. In the equivalent experiment with *Sc hmpA1*, the holo NsrR-*hmpA1* complex corresponded to 100% relative intensity (Fig. S4B†). Thus, despite the reduced affinity, the binding of holo NsrR remained largely specific for cognate promoter sequences.

**Fig. 5:**
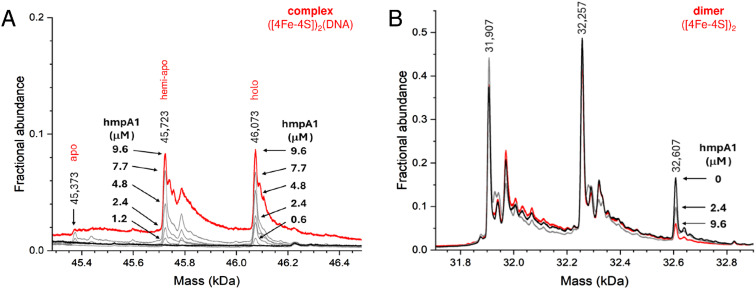
Formation of NsrR-*hmpA1* complexes with heterogeneous NsrR. (A) Deconvoluted mass spectra at selected concentrations of *hmpA1* DNA showing the formation of NsrR-*hmpA1* complexes from heterogenous NsrR samples (black line, 8 μM [4Fe–4S], 11.4 μM protein, ∼70% loaded). At higher concentrations of *hmpA1* hemi-apo and holo NsrR-*hmpA1* complexes were observed (red line); see Fig. S3B† for annotation of adducts. At low concentrations (≤3 μM) there was a clear preference for holo NsrR dimers (grey lines, as annotated). Apo NsrR exhibited a negligible affinity for DNA, consistent with previous observations. (B) Dimeric region of the spectrum during the same titration, showing the decline of holo NsrR as the titration proceeded. Minor changes in the amount of uncomplexed hemi-apo NsrR dimers were also observed. Proteins and complexes were ionised from 100 mM ammonium acetate, pH 8, with positive mode ESI.†

Finally, to determine if other NsrR species might be capable of binding DNA, we repeated the native MS titrations using the heterogeneous, as-isolated NsrR sample (Fig. 5). Complexes of *hmpA1* with hemi-apo or holo NsrR readily appeared, but at lower concentrations of DNA (0.58 μM *hmpA1*) there was a clear preference for holo NsrR over hemi-apo NsrR (Fig. 5A). To determine the relative affinities of hemi-apo and holo NsrR for *hmpA1*, the fractional abundances of free and DNA-complexed forms were analysed, as described above, using a simple equilibrium binding model (Fig. S5†). The resulting fit of the data revealed that binding of hemi-apo NsrR to *hmpA1* was significantly weaker (estimated to be ∼*K*_d_ = ∼40 μM) than that of the holo form (*K*_d_ = ∼3 μM, as above).

Thus, native MS data clearly demonstrated that holo NsrR preferentially binds *hmpA1* DNA with a relative affinity ∼10 times higher than that for hemi-apo NsrR. Dimeric apo NsrR exhibited negligible DNA binding, consistent with previous observations from EMSA.^22^

### SPR kinetics of binding to *hmpA1* and *hmpA2* promoters

EMSA experiments previously indicated that binding of holo NsrR occurs with different affinities to the promoters of the three NsrR-regulated genes in *S. coelicolor*.^22^ To gain a more quantitative understanding of binding of holo NsrR to *hmpA1* and *hmpA2* promoters, we again utilised ReDCaT SPR, but with appropriate modifications to enable measurement of kinetic parameters that permit determination of association and dissociation rate constants, and hence binding affinities.^45,47^

**Fig. 6:**
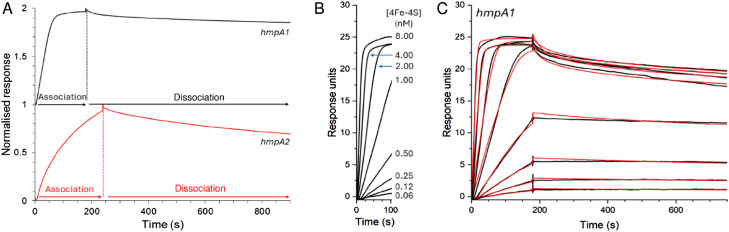
SPR kinetics of NsrR-DNA complex formation and dissociation. (A) Comparison of association and dissociation phases for 2 nM [4Fe–4S] NsrR binding to *hmpA1* (black line) and *hmpA2* (red line) promoter sequences using anaerobic SPR buffer. (B) Association phase form *hmpA1* promoter at varying concentrations of [4Fe–4S] NsrR, as indicated. (C) Full association and dissociation phases of data shown in B (black line), together with fits to a bivalent analyte model (red line), giving association and dissociation rate constants and binding affinity, see Table 1. For *hmpA2* analysis, see Fig. S6.† Analysis temperature was 25 °C.

The interaction of NsrR with the immobilised *hmpA1* or *hmpA2* sequences gave sensorgrams with clear association and dissociation phases (Fig. 6A). A bivalent analyte model was required for analysis of the data (see Methods). This encompassed the primary interaction of analyte and ligand molecules, and a secondary interaction with additional immobilised ligand molecules, consistent with native MS titrations (see above).

Use of the bivalent analyte model gave satisfactory fits to the data, with an association rate constant, *k*_a_ = 2.04 × 10^7^ M^−1^ s^−1^, dissociation rate constant, *k*_d_ = 24.05 × 10^−3^ s^−1^, and an equilibrium dissociation constant, *K*_d_ = 1.18 nM for the binding of holo NsrR to *hmpA1* (Table 1). The *K*_d_ derived from kinetic experiments was comparable to that derived from the equilibrium experiments described above (see Fig. 3). For *hmpA2*, analyses yielded *k*_a_ = 2.26 × 10^6^ M^−1^ s^−1^, *k*_d_ = 57.93 × 10^−3^ s^−1^ and a *K*_d_ of 25.6 nM (see Fig. S6,† and Table 1). These *K*_d_ values are qualitatively consistent with the previously reported EMSA data for each promotor for which binding to *hmpA1* was significantly tighter.^22,34^ Observed secondary binding events were qualitatively consistent with the advent of non-specific DNA binding in previously reported EMSAs.^34^

**Table 1:** Kinetic and thermodynamic parameters for binding of holo NsrR to DNA

DNA	Primary binding			Secondary binding		
	*k*_a_ (M^−1^ s^−1^)	*k*_b_ (s^−1^)	*K*_d_ (nM)	*k*_a_ (M^−1^ s^−1^)	*k*_b_ (s^−1^)	*K*_d_ (nM)
*hmpA1*	2.04 × 10^7^	24.05 × 10^−3^	1.18 (±0.2)	49.9 × 10^3^	2.34 × 10^−3^	47 (±6.2)
*hmpA2*	2.26 × 10^6^	57.93 × 10^−3^	25.6 (±0.7)	18.1 × 10^3^	5.09 × 10^−3^	281 (±7.4)

Overall, holo NsrR was found to associate with *hmpA2* an order of magnitude more slowly than it does with *hmpA1*, which, in combination with a ∼2.5 times faster dissociation phase, leads to a ∼25-fold less stable complex with *hmpA2* compared to that observed with *hmpA1*.

### Nitrosylation of NsrR-DNA complexes

Although DNA binding is clearly affected by the presence of ammonium acetate, the above data illustrate the power of parallel SPR and native MS measurements to provide novel insight into the different complexes present in solution. Attempts were made to monitor the NO-induced dissociation of holo NsrR *hmpA1* complexes by SPR, but these were unsuccessful despite flushing the SPR system with anaerobic buffers. We note that the concentration of NO in anaerobic solutions declines slowly with time, but if O_2_ is present, it decays extremely rapidly.^57,58^ While the anaerobic half-life of aqueous NO (*t*_½_ = 8 min) would be compatible with SPR analysis, we conclude that ingress of O_2_ in the flow path of the SPR instrument is sufficient to destroy the injected NO (≈2 μM) prior to it reaching the immobilised holo NsrR-DNA complexes.

Unlike the SPR system, the native MS setup utilises PEEK tubing and gas-tight syringes to limit O_2_ ingress into the system once it has been flushed with anaerobic buffer. We have previously shown that the nitrosylation of NsrR is extremely rapid and that slow NO-releasing reagents (*e.g.* NONOates) can be used, *in situ*, to study the reaction of [4Fe–4S] NsrR with NO by native MS.^37,38^ Under these conditions, NO availability limits the rate of reaction, enabling an effective thermodynamic titration of samples with NO. Furthermore, using ^57^Fe/^34^S substituted [4Fe–4S] NsrR, we have previously demonstrated that the +30 Da peak results from the mono-nitrosylation of the NsrR cluster, and that the intensity of this peak decays away above ∼3 NO per cluster as higher order NO complexes are formed.^38^

**Fig. 7:**
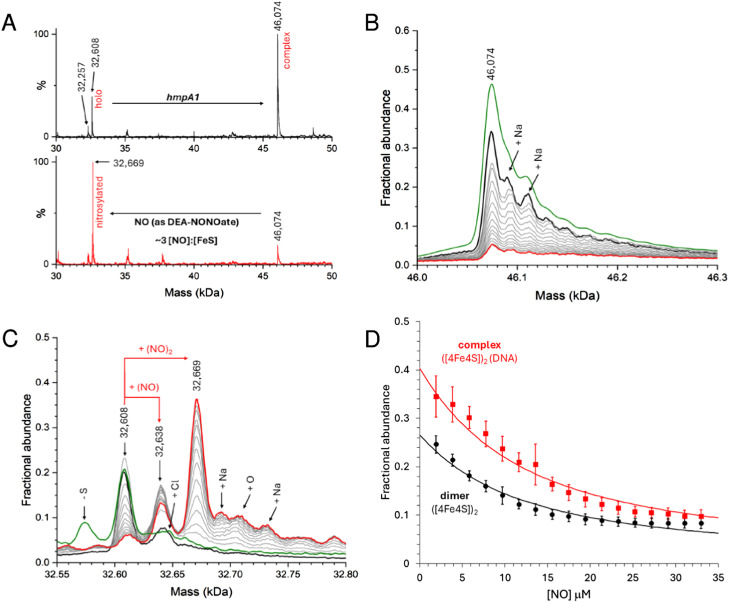
Nitrosylation of NsrR-*hmpA1* complex probed by native MS. (A) Comparison of NsrR-*hmpA1* complexes before (black line) and after (red line) the addition of NO (as DEA NONOAte). Spectra of NsrR-*hmpA1* complex (B) and dimeric NsrR regions (C) are shown in more detail as the *in situ* nitrosylation proceeds. Following the addition of NONOate (black lines) the spectra gradually change as NO is released (grey lines, increments of ∼0.2 [NO] : [4Fe–4S]). After ∼3.5 [NO] : [4Fe–4S] most of the NsrR-*hmpA1* complex was dissociated (red lines). The green line in (C) shows a comparable sample in the absence of DEA NONOate. (D) Average (*n* = 4) fractional abundance for the NsrR-*hmpA1* complex (red squares) and holo NsrR dimers (black circles) as a function of the [NO] concentration. Error bars represent standard deviations. Solid lines represent fits using a simple equilibrium model (see Methods). Samples contained 8 μM [4Fe–4S], 8 μM *hmpA1* DNA.

Here we used DEA NONOate to follow the *in situ* nitrosylation of NsrR-DNA complex by native MS, where the ionization of dimeric species was optimised. Samples prepared with 8 μM [4Fe–4S] NsrR (4 μM dimer) and 8 μM dsDNA *hmpA1* were found to contain holo NsrR-DNA complexes, together with unbound holo NsrR (∼40% relative intensity). The addition of 15 μM DEA NONOate resulted in the loss of NsrR-DNA complexes, and concomitant appearance of unbound nitrosylated NsrR species after 35 min, equating to ∼3 NO per [4Fe–4S] (Fig. 7A). More detailed views of relevant species in the non-DNA bound and bound regions are shown in Fig. 7B and C, respectively, and broader mass range spectra are shown in Fig. S7A and B.†

Control experiments containing an equivalent volume of carrier solution, but lacking DEA NONOate, had little effect on the stability of NsrR-DNA complexes over the same time course (Fig. S7C–F†). Once the reaction was initiated, clear evidence for the formation of mono- and dinitrosyl-holo NsrR was obtained, with no evidence for further iron-nitrosyl formation, (*e.g.* DNIC, RRE, RBS), or a significant increase in the abundance of hemi-apo or apo NsrR. Thus, the data indicate that mono- or di-nitrosylation of the NsrR dimer caused a loss of DNA binding (Fig. S7A and B†).

During *in situ* nitrosylation, the decay of NONOate to yield free NO represents the rate-limiting step of all subsequent downstream reactions. By careful calibration of NO release, precise ratios of NO per [4Fe–4S] cluster or NsrR dimer could be determined, enabling us to follow the decay of unbound and DNA-complexed NsrR species in response to increasing NO, as well as the appearance of nitrosylated NsrR cluster species during the course of the reaction. The fact that mono-nitrosylated NsrR in complex with DNA was not observed suggested either that the nitrosylation reaction is concerted so that such a species immediately reacts with further NO, or that binding of a single NO molecule to the NsrR dimer disrupts DNA binding. The detection of unbound mono-nitrosylated NsrR strongly favours the latter conclusion.

Data describing the decay of the DNA-bound and unbound NsrR dimer were fitted using an equilibrium model (see Methods) describing the simultaneous mono-nitrosylation of unbound and DNA-complexed holo NsrR, Fig. 7D, from which a *K*_d_ for NO-binding was determined as 4.8 ± 0.5 μM. This was independent of whether or not the protein was bound to DNA (*i.e.* satisfactory fits of both datasets resulted in the same *K*_d_). In addition, the appearance of unbound DNA could also be monitored and fitted using the same *K*_d_, Fig. S8.†

The inability of mono-nitrosylated NsrR to bind DNA is compatible with the much lower affinity of hemi-apo NsrR for DNA, and the lack of DNA binding observed for the D8A holo NsrR variant, in which one of the cluster ligands was replaced by a non-coordinating residue.^34^ It is also consistent with the lack of concerted binding of NO observed for the non-DNA bound form.^37^ Thus, it is likely that di-nitrosylated NsrR is derived from the free mono-nitrosyl species, rather than from the sequential interaction of two NOs with the holo NsrR-DNA complex.

The interaction of NO with NsrR-*hmpA2* complexes was also investigated. Following the addition of *hmpA2*, approximately equal relative intensities of the free holo NsrR and NsrR-DNA complexes was observed (Fig. S9A,† black like). This behaviour is significantly different to NsrR in the presence of *hmpA1* and presumably reflects the reduced affinity of NsrR for the *hmpA2* promoter (compare Fig. S9A† to 6A). During *in situ* nitrosylation, we again observed a loss of NsrR-DNA complexes, and concomitant appearance of unbound nitrosylated NsrR species. During the reaction, mono-nitrosylated-NsrR in complex with DNA was not observed, while unbound mono- and di-nitrosylated NsrR species accumulated, as was the case of NsrR-*hmpA1*. Satisfactory fits to the NsrR-*hmpA2* data were obtained using the same simple equilibrium model used to fit NsrR-*hmpA1* data (Fig. S9B†), yielding a similar *K*_d_ of 2.7 ± 0.4 μM. Thus, we conclude that mono-nitrosylation of a single [4Fe–4S] cluster within the NsrR dimer is sufficient to disrupt an otherwise stable holo NsrR-DNA complex.

## Discussion

NsrR senses and responds to the endogenous production of NO *via* a mechanism involving its direct binding to the iron-sulfur cluster.^8,22,25,37^ Most, but not all, of this NO is formed endogenously by the interaction of nitrite with cytoplasmic nitrate reductases.^8–11^ Previously, a range of biophysical approaches showed that NsrR contains a [4Fe–4S] cluster, and that this form of the protein recognises an imperfect inverted repeat located in DNA sequences upstream of NsrR-regulated genes, thus repressing transcription.^22,32–34^ Where studied, many of the genes regulated by NsrR are involved in mitigating nitrosative stress, with the principal gene encoding a flavohemoglobin (*hmp*) that converts NO to NO_3_^−^ under (micro)aerobic conditions.^22,27,28,59,60^

Previously, we have shown that [4Fe–4S] NsrR rapidly reacts with NO *via* a multiphasic, non-concerted, process that leads to the loss of DNA binding and concomitant formation of several different iron-nitrosyl species at distinct [NO] : [4Fe–4S] ratios.^37^ Through a combination of nuclear resonance vibrational spectroscopy, LC-MS, and, in particular, native MS, we were able to identify many of the intermediates and products in this general nitrosylation process.^38,39,41^ This previous work provided a remarkable insight into the nitrosylation process, permitting identification of protein-bound mono- and di-nitrosyl [4Fe–4S] complexes ([4Fe–4S]-NO and [4Fe–4S]-(NO)_2_, respectively), dinitrosyl iron complexes (DNIC, [Fe(NO)_2_(Cys)_2_]), and species similar Roussin's red esters (RRE, [Fe_2_(NO)_4_(Cys)_2_]) and Roussin's black salts (RBS, [Fe_4_(NO)_7_(S)_3_]). However, it did not identify the key sensing step, *i.e.* at what point in the reaction loss of DNA binding occurs.

EMSAs are often used to assess protein-DNA binding, but there are some inherent disadvantages that makes them potentially unreliable in certain circumstances (reviewed inrefs. 61 and 62). Thus, absolute binding affinities are better determined by more sensitive analytical methods such as surface plasmon resonance (SPR). SPR is well suited to the study of iron-sulfur transcriptional regulators as measurements can be performed rapidly, limiting the potential for cluster degradation during analysis.^46^ Together with native MS observations, these two techniques provide a much greater insight into the exact species interacting with the DNA than is available from SPR or EMSAs alone.^42,46^

SPR data for the binding of reconstituted holo NsrR to *hmpA1* revealed a high-affinity (nanomolar *K*_d_) site. SPR also revealed that the apparent affinity of holo NsrR for DNA is reduced under the buffer conditions required for native MS measurements due to competition with acetate. Despite this reduced apparent affinity, holo NsrR remained capable of discriminating between cognate *Streptomyces hmpA1* and non-cognate *Croynebacteria hmp* promoter sequences.^56^ Thus, a combination of native MS and SPR unambiguously demonstrated that holo NsrR is the key DNA-binding species in solution, consistent with crystal structures.^33^ Native MS also demonstrated that, under certain circumstances, hemi-apo NsrR is capable of binding to DNA, but that it does so with an affinity >10-fold lower than that of holo NsrR. Apo NsrR did not interact with DNA, consistent with earlier EMSA observations.^22^

Previous EMSA observations also suggested that holo NsrR binds to the *hmpA2* promoter with a lower affinity compared to *hmpA1*.^22^ Here we suitably modified the ReDCaT application of SPR to enable kinetic experiments, which could provide more detailed understanding of holo NsrR binding behaviour.^45–47^ Kinetic SPR data sets were best described by a bivalent analyte model, revealing rapid association of holo NsrR with *hmpA1* (*k*_a_ = ∼2 × 10^7^ M^−1^ s^−1^), and relatively slow dissociation (*k*_d_ = ∼24 × 10^−3^ s^−1^), giving a *K*_d_ of ∼1 nM. In contrast, holo NsrR associated with the *hmpA2* promoter an order of magnitude more slowly than with *hmpA1* (∼3 × 10^6^ M^−1^ s^−1^) and dissociated ∼2.4 times faster (∼58 × 10^−3^ s^−1^) giving a *K*_d_ of ∼26 nM. It was recently shown that the central region of the NsrR promoter sequence is not a key determinant for NsrR binding.^33^ It is likely that structural differences in the promoter sequence ‘read’ by holo NsrR govern DNA binding, with loss or gain of favourable protein-DNA interactions resulting in alterations in the association and dissociation kinetics.

We have previously used native MS, in combination with *in situ* nitrosylation, to successfully follow the full nitrosylation of holo NsrR.^38^ Here, we have again used *in situ* nitrosylation but focused on a narrow range of [NO] : [4Fe–4S] ratios, and, importantly, with NsrR present as the holo NsrR-*hmpA1* complex, enabling the direct detection of reaction of the NsrR cluster while on DNA. The data demonstrated that the interaction of NO with holo NsrR-*hmpA1* complex resulted in the formation of unbound mono-nitrosylated NsrR (containing one [4Fe–4S] and one [4Fe–4S](NO) cluster per dimer) and unbound DNA. Thus, the binding of a single NO to one of the two [4Fe–4S] clusters of the NsrR dimer is sufficient to disrupt DNA binding, indicating that the NsrR-DNA complex is exquisitely sensitive to NO once it reaches a potentially harmful level.

While the concentration of NO produced endogenously in the bacterial cytosol during nitrate reduction has not been determined directly, recent literature indicates that cellular concentrations of NO are an order of magnitude lower than previously thought and rarely higher than low micromolar levels.^63–65^ Thus, the physiological significance of *in vitro* nitrosylation experiments conducted with NO concentrations significantly higher than those present *in vivo* is unclear. There is a risk that such studies, although often interesting, may not be physiologically relevant. The data presented here show that, in the case of NsrR, NO sensing occurs upon the binding of a single NO, and does not involve protein-bound iron-nitrosyls similar to DNIC, RRE or RBS. Thus, the previously reported full nitrosylation reactions of the NsrR cluster, involving ∼8 NO per cluster, likely fall into the category of interesting but not physiologically relevant, at least with respect to *hmpA1* and *hmpA2* promoter binding. The affinity of NO for the [4Fe–4S] cluster is estimated to be in the low micromolar range, indicating that NsrR is insensitive to the low (pM – nM) concentrations of NO that are estimated to be present *in vivo* due to background levels of nitrate reductase-catalysed reduction of nitrite to NO.^64^

A further NO molecule was able to bind to mono-nitrosylated NsrR dimers, to presumably give NsrR dimers containing two [4Fe–4S](NO) clusters (di-nitrosylated NsrR). NsrR dimers containing one unreacted cluster and one [4Fe–4S](NO)_2_ cluster may also be formed. Previous studies demonstrated that the cluster degradation begins at [NO] : [4Fe–4S] ratios ≥2, resulting in sulfide oxidation and formation of protein-bound DNIC, RRE and RBS-like species.^38,39,66^ No evidence of such species was observed in these experiments, consistent with mono-nitrosylation of [4Fe–4S] clusters.

Recently, 3 : 1 site-differentiated [4Fe–4S] model complexes have been reported that, upon exposure to NO, resulted in the dissociation of the unique ligand to give a mono-nitrosylated form of the [4Fe–4S] cluster.^53,67^ A mono-nitrosylated [4Fe–4S] model complex with a physiologically relevant coordination comprising three sulfur and one carboxylate ligand was also shown to form upon introduction of NO, again with displacement of the carboxylate.^53^ Furthermore, nitrosylation reactions of D8A and D8C variants of NsrR were significantly affected, consistent with the importance of the labile Asp ligand for control of the reaction with NO.^53^

As previously proposed, and supported by data from the model cluster studies described above,^34,53,67^ the first NO molecule likely displaces Asp8 from the cluster of one subunit of the holo NsrR dimer, generating mono-nitrosylated NsrR, [4Fe–4S](NO). Displacement of Asp8 from the cluster effects a conformational change in the wHTH domain, decreasing the affinity of dimeric NsrR for DNA, resulting in dissociation. As discussed above, reaction of a second NO most likely occurs at the unreacted cluster of the NsrR dimer, generating a second mono-nitrosylated cluster. Further additions of NO result in binding of a second NO molecule to each cluster.

From a biological perspective, the above observations suggest that the *hmpA1* promoter will be occupied by holo NsrR in the absence of NO, repressing transcription. Under microaerobic/anaerobic conditions the half-life of cytoplasmic NO will increase, leading to mono-nitrosylation of holo NsrR, loss of DNA binding and de-repression of transcription. Due to the reduced affinity of *hmpA2* for holo NsrR, this gene is probably rarely subject to full NsrR-mediated repression. This is consistent with previous ChIP-seq data, in which NsrR was 20 times more likely to be isolated with *hmpA1* promoter fragments than with *hmpA2* fragments.^22^

Where studied *in vivo*, *hmpA1* expression is linked to the presence of nitrate reductase and NO_2_^−^, and it is an important factor for mitigating the effects of NO.^3,31^ We note that mRNA transcripts of *nsrR* remain relatively constant throughout growth of *S. coelicolor* in liquid medium. Transcripts of *hmpA1* are also present, but decline in abundance towards stationary phase, indicative of NsrR repression.^68^ In contrast, transcripts of *hmpA2* are ∼40 times lower than *nsrR* or *hmpA1* transcripts and remain constant throughout growth.^68^ We also note that *hmpA2* under its natural promoter was unable to compensate for the loss of *hmpA1* in Δ*hmpA1* mutants, while Δ*hmpA1::hmpA1* complementation restored a wild type-like phenotype.^3^ Together, these observations suggest the *hmpA2* may be controlled by factors other than NsrR, or that *hmpA2* is redundant. NsrR regulates its own expression, and it was previously shown that the affinity of holo NsrR for the *nsrR* promoter is lower than that for *hmpA1*.^22^ Although we have not studied this further here, this is consistent with an intermediate binding affinity that ensures that NsrR is produced so that it can downregulate *hmpA1* expression until nitrosative stress occurs.^22,69^

Once expressed, HmpA1 will lower cytoplasmic concentrations of NO, reinstating NsrR-mediated repression, suggesting NO binding may be reversible. Where NO persists long enough, binding of further NO molecules to an already nitrosylated NsrR cluster likely triggers cluster disassembly and concomitant formation of protein-bound iron-nitrosyls (DNIC, RRE- or RBS-like species) and apo NsrR. Protein bound iron-nitrosyls (RRE- or RBS-like species) may be subject to disassembly *in vivo* due to high concentrations of low molecular weight thiols (mycothiol in *Streptomyces*) to give low molecular weight DNIC species.^70–73^ Subsequent detection of NO by the heme-based DevS/R two component system can then attenuate nitrate reductase expression,^30^ which would reduce NO production. In considering why NsrR-mediated repression has evolved to be so sensitive to increasing levels of cytoplasmic NO, it is noteworthy that NO detoxification is upregulated early on, *i.e.* with the binding of the first NO, and does not require the accumulation of NO and consequent formation of iron-nitrosyls on NsrR. Thus, the scope for widespread damage to other Fe–S clusters (and other metallocofactors) is limited.

**Fig. 8:**
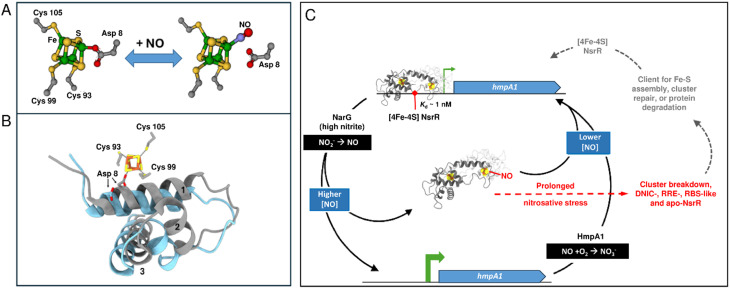
Proposed model of NO sensing by holo NsrR. (A) NO binds to the one of the clusters present in holo NsrR, displacing Asp8 to giving a mono-nitrosylated holo NsrR. (B) Displacement of Asp8 by NO likely results in the repositioning of the recognition helix (helix 3), disrupting DNA binding (blue, PDB: 5N08 ref. 34). For comparison, the wHTH domain of NsrR-DNA complex is shown in grey (PDB: 7B0C). (C) Scheme summarizing the mechanism of NsrR NO sensing and regulation. Holo NsrR binds to the promoter sequence upstream of *hmpA1* (encoding a flavohemoglobin NO dioxygenase), repressing expression. Endogenous NO is produced by NarG when cytoplasmic NO_2_^−^ concentrations are elevated. Detection of NO by the [4Fe–4S] cluster of NsrR leads to mono-nitrosyl (and di-nitrosyl) holo NsrR dimers and a loss of DNA binding, allowing the expression of *hmpA1* in an NO-dependent manner. HmpA1 converts NO to back to NO_2_^−^, lowering the cytoplasmic NO concentration, reinstating holo NsrR-mediated repression. During prolonged nitrosative stress (shown in red) the [4Fe–4S] breaks down, becoming DNIC, RRE, RBS-like and apo-NsrR species.^38,53,70^ These forms of NsrR (shown in grey) may be clients for FeS cluster assembly, repair, or a target for protein degradation.^70,73,74^ Repaired (or replaced) [4Fe–4S] NsrR may re-bind the *hmpA1* promoter if the cytoplasmic NO concentration is low enough.

In summary, a combination of SPR and native MS have provided novel insight into the nature of NsrR-DNA complexes and how they subsequently respond to NO, with clear implications for *in vivo* function. Holo NsrR rapidly associates with the *hmpA1* promoter to form a tightly bound complex. When NO levels increase to low micromolar concentrations, a single NO molecule reacts with the cluster on one side of the NsrR dimer, generating a mono-nitrosylated form. Like hemi-apo NsrR, the mono-nitrosylated dimer has low affinity for DNA, alleviating transcriptional repression of *hmpA1*. Subsequent detoxification of NO by the action of HmpA1 would lower the cytoplasmic NO concentration, leading to dissociation of NO from [4Fe–4S] NsrR and re-binding of NsrR to DNA. Sensing NO through binding of a single molecule to NsrR means that NO detoxification is switched on as soon as is practicable, limiting the possibility of cellular damage. Under extended or severe nitrosative stress, degradation of the NsrR cluster, resulting in iron-nitrosyl species, would occur, precluding re-binding to DNA without intervention of cluster repair or assembly. This proposed model for NO sensing is summarised in Fig. 8.

The data presented here, revealing exquisite sensitivity of the NsrR-DNA complex to low micromolar concentrations of cytoplasmic NO, are consistent with *in vivo* observations of NO levels present in the bacterial cell.^8,64^ The mechanism by which the binding of a single NO could effect a conformational change sufficient to abolish DNA binding very likely involves the displacement by NO of the carboxylate side chain of Asp8 as a ligand to the [4Fe–4S] cluster. This is predicted to disconnect the cluster from the wHTH domain of the other subunit in the dimer, allowing the recognition helix of the wHTH domain to adopt a conformation incompatible with binding DNA.^34^

Finally, the subsequent formation of NsrR-bound iron-nitrosyls, such as DNIC, RRE and RBS species, at higher levels of NO is clearly not required to alleviate NsrR-mediated repression of transcription. Thus, these species are not relevant to the on/off switch controlling *hmpA1* expression. However, they may have physiological relevance under severe nitrosative stress where their formation upon cluster degradation would preclude re-association of the nitrosylated NsrR with DNA, necessitating cluster repair/re-insertion or new protein synthesis in order to re-establish NsrR-mediated repression.

## Data availability

Data supporting the conclusions of this study are available in the main paper with additional experimental data given in the ESI.† All data are available from the corresponding author upon request.

## Author contributions

J. C. C.: conceptualization, investigation, formal analysis, writing – original draft. N. L. B.: conceptualization, funding acquisition, supervision, writing – review and editing.

## Conflicts of interest

The authors have no conflicts to declare.

## Supplementary Material

SC-OLF-D4SC04618H-s001
