# Supplementary material for: Binding of a single nitric oxide molecule is sufficient to disrupt DNA binding of the nitrosative stress regulator NsrR
Source: Chem Sci. 2024 Oct 15;15(45):18920–32. doi: 10.1039/d4sc04618h (PMC11500311; doi:10.1039/d4sc04618h)
Supplement: SC-015-D4SC04618H-s001 [file SC-015-D4SC04618H-s001.pdf]

Supplementary Information (SI) for Chemical Science.
This journal is © The Royal Society of Chemistry 2024

Supporting Information

Binding of a Single Nitric Oxide Molecule is Sufficient to Disrupt DNA Binding of the Nitrosative Stress Regulator NsrR

.

Jason C. Crack and Nick E. Le Brun

Supporting Tables

Table S1. Oligonucleotides used for SPR in this study. ReDCaT sequence is shown in red.

Name	Sequence (5'→3')
**ReDCaT_F**	Biotin//GCAGGAGGACGTAGGGTAGG
**ReDCaT_R**	CCTACCCTACGTCCTCCTGC
***hmpA1*_F**	TAAAACACGAATATCATCTACCAATTAAG
**ReDCaT *hmpA1*_R**	CTTAATTGGTAGATGATATTCGTGTTTTACCTACCCTACGTCCTCCTGC
**hmpA2_F**	GAAAACAAGCATCTGAGATCCCAGTTCGG
**ReDCaT *hmpA2*_R**	AAGAACTGGGATCTCAGATGCTTGTTTTCCCTACCCTACGTCCTCCTGC

Table S2. Oligonucleotides used for native MS in this study.

Name	Sequence (5'→3')	Mass (Da)	$\varepsilon_{260\ nm}$ (mM^{-1} cm^{-1})
***hmpA1*_F**	AACACGAATATCATCTACCAAT	6655.38	223.5
***hmpA1*_R**	ATTGGTAGATGATATTCGTGTT	6810.43	220.3
***hmpA2*_F**	AACAAGCATCTGAGATCCCAGTT	7016.59	227.8
***hmpA2*_R**	AACTGGGATCTCAGATGCTTGTT	7069.59	219.1
**Cro *hmp*_F**	GGATTTAATCTAATTAAATAAATCCCAGGA	9221.04	311.8
**Cro *hmp*_R**	TCCTGGGATTTATTTAATTAGATTAAATCC	9184.96	293.8

Table S3. Predicted and observed masses for all NsrR species.

Species	Predicted mass[a] (Da)	Observed mass[b] (Da)	ΔMass[c] (Da)
**$(NsrR)_2$**			
[apo]	31,909	31,907	-2
[hemi-apo]	32,259	32,257	-2
[holo]	32,608	32,608	0
hmpA1::[apo]	45,375	45,373	-2
hmpA1::[hemi-apo]	45,723	45,723	0
hmpA1::[holo]	46,075	46,074	-1
$(hmpA1)_2$::[holo]	59,540	59,541	+1
hmpA2::[holo]	46,695	46,694	-1
Cg hmp::[holo]	51,015	51,015	0
**Nitrosylated $(NsrR)_2$**			
[holo]	32,608	32,608	0
[holo](NO)	32,639	32,639	0
[holo]$(NO)_2$	32,669	32,670	+1
[hemi-apo]	32,259	32,258	-1
[hemi-apo](NO)	32,289	32,288	-1
[hemi-apo]$(NO)_2$	32,319	32,319	0

[a]The predicted mass depends on the cluster/cluster fragment charge because binding is assumed to be charge compensated [1]. Cluster charge states are as observed previously [2, 3].
[b]The average observed mass is derived from at least two independent experiments, with standard deviation of ± 1 Da. Deviation of - 2 Da are indicative of disulfide bond formation.
[c]The difference between the average observed and predicted masses.

Supporting Figures

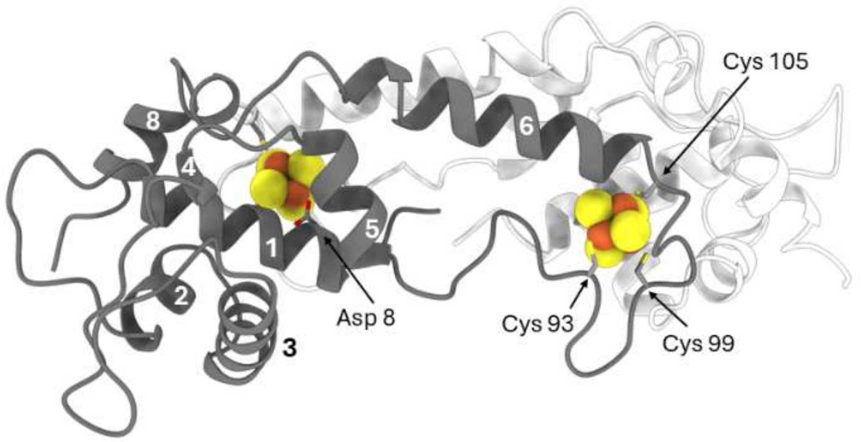

Figure S1. Crystal structure of [4Fe-4S] NsrR (PDB 5NO7 [4]). *S. coelicolor* NsrR is a homo-dimer, composed of elongated monomeric subunits (grey and white, respectively). Each subunit contains an N-terminal wHTH DNA-binding domain (helices 1 – 3)**,** long dimerization helix (helix 6), and a C-terminal loop (between helices 5 & 6) than binds the [4Fe-4S] cluster. The cluster is an integral part of the inter-subunit connection network that correctly positions the recognition helices for DNA binding (see Fig. 1B).

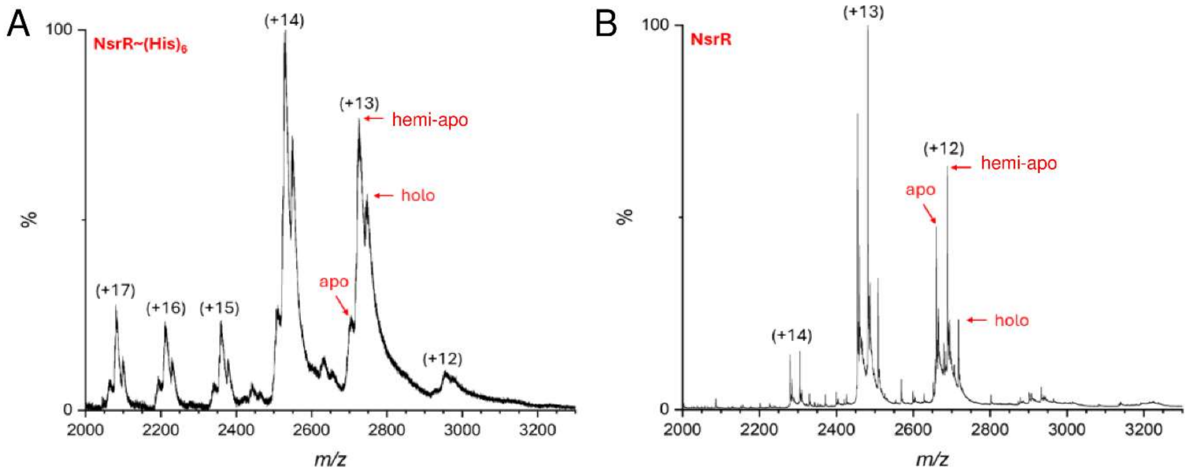

Figure S2. Native MS of His-tagged and non-tagged [4Fe-4S] NsrR. Comparison of *m/z* spectra for **A)** His-tagged NsrR and **B)** non-tagged [4Fe-4S] NsrR in the dimeric region. Charge states and peaks originating from apo, hemi-apo and holo NsrR are indicated. Samples contained 8 μM [4Fe-4S] and were ionised from 100 mM ammonium acetate, pH 8.

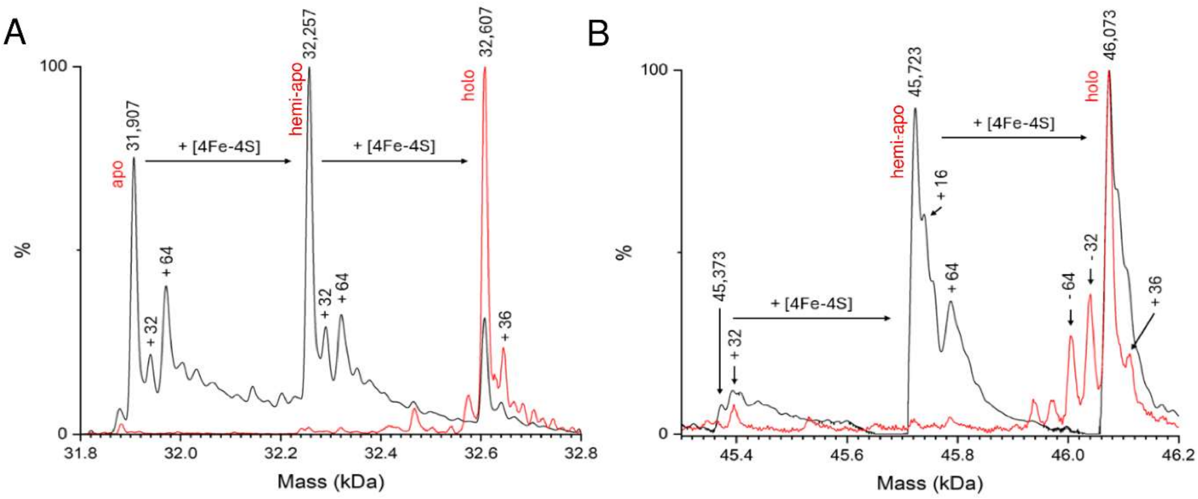

Figure S3. Further detail of native MS data for [4Fe-4S] NsrR and NsrR-*hmpA1* complexes. Deconvoluted spectra for as-isolated (black line) and reconstituted [4Fe-4S] NsrR (red line) **A)** before and **B)** after the addition of *hmpA1* DNA. **A)** shows the dimeric region of the spectrum, **B)** shows the region corresponding to NsrR-DNA complexes. Peaks originating from apo, hemi-apo and holo NsrR are indicated. Samples contained 8 μM [4Fe-4S] and were ionised from 100 mM ammonium acetate, pH 8.

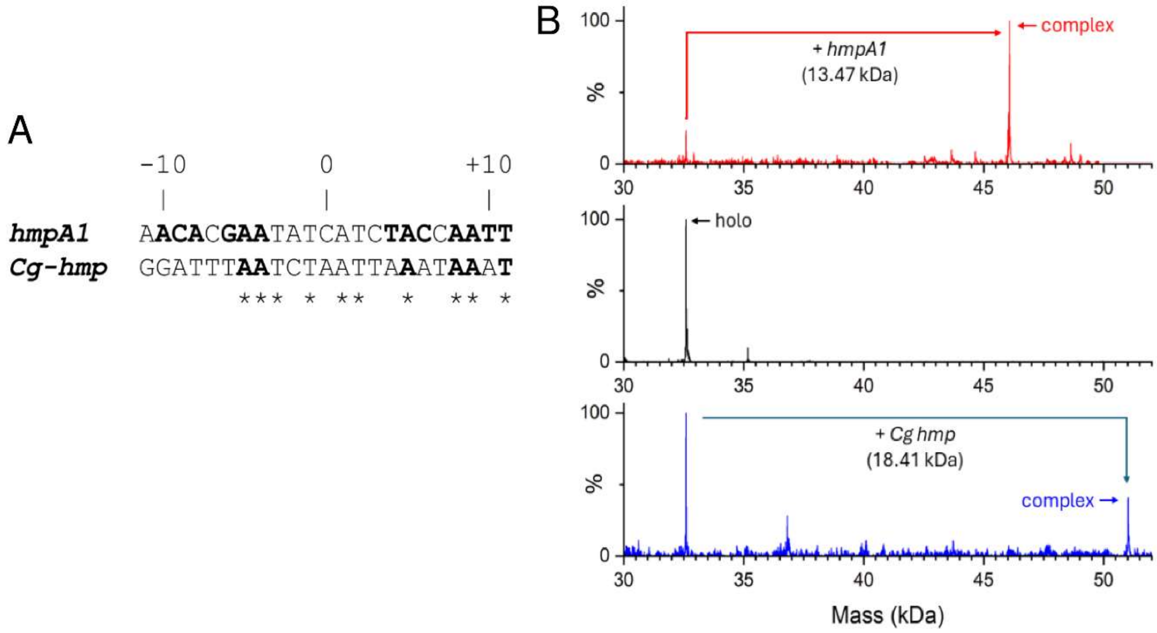

Figure S4. NsrR can discriminate between *hmpA1* and non-cognate *Cg-hmp* DNA. A) Sequences of *hmpA1* and *Cg-hmp* promoter DNA used in this study. Known protein-base interactions are shown in bold for the *hmpA1* promoter; bases occupying an identical position in the *Cg-hmp* promoter that may similarly interact with NsrR are shown in bold. The *Cg-hmp* promoter shares ~43% identity to *hmpA1*, with identical bases indicated by an asterisks. **B)** Dimeric NsrR (black line) can discriminate between cognate *hmpA1* (red line) and the non-cognate *Cg hmp* sequences (blue line), as judged from native MS data. Uncomplexed NsrR is more abundant in the presence of *Cg-hmp* than *hmp1A* promoter DNA, indicating sequence specificity is preserved during native MS experiments. NsrR (8 μM [4Fe-4S]) was treated with a twofold excess of DNA (16 μM DNA) prior to ionization.

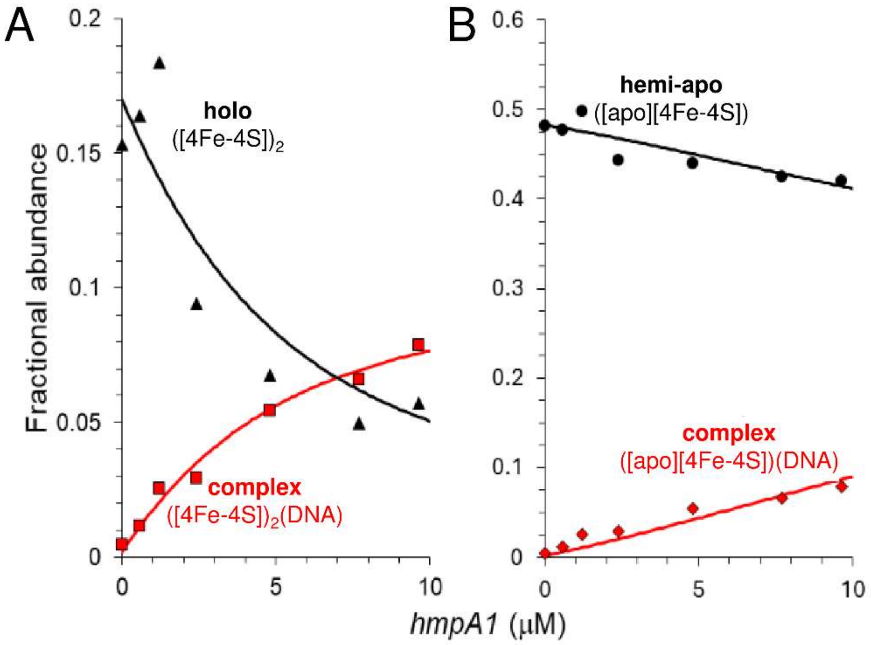

Figure S5. Formation of [4Fe-4S] NsrR-*hmpA1* complexes with heterogeneous NsrR. Annotated plots of relative intensity for **A)** holo NsrR and **B)** hemi-apo NsrR species as a function of the *hmpA1* concentration. Solid lines show fits of the data to a simple sequential binding model. This yielded a K_d of ~3 µM for the holo NsrR-*hmpA1* complex. For the hemi-apo NsrR complex, binding was weak and so only the first part of the binding was detected, from which a K_d of ~41 µM was estimated.

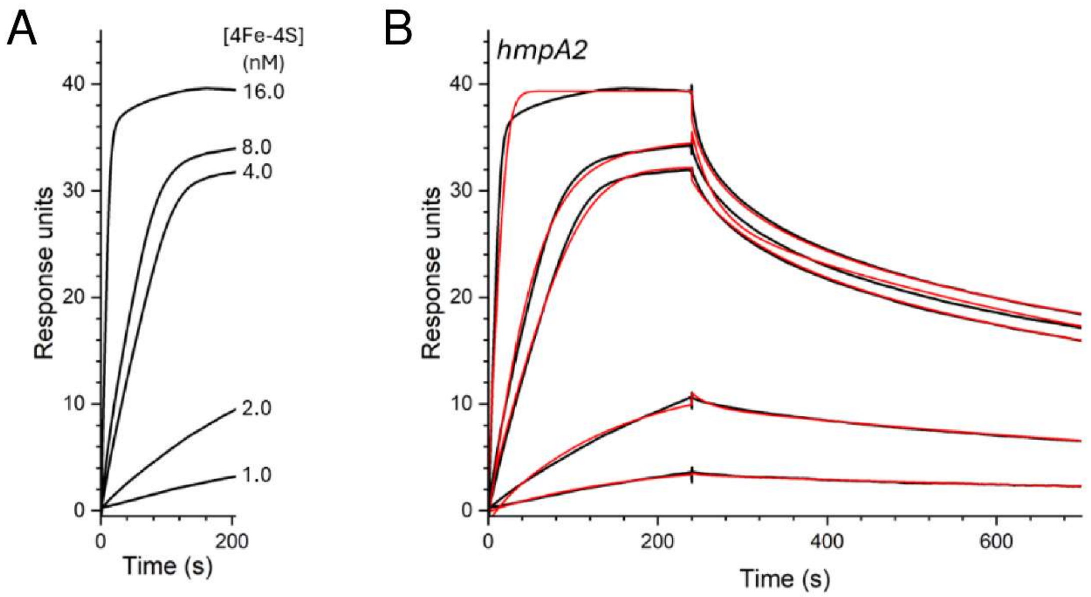

Figure S6. Kinetics of NsrR-*hmpA2* complex formation and dissociation as measured by SPR. A) Association phase form *hmpA2* promoter at varying concentrations of [4Fe-4S] NsrR, as indicated. **B)** Full association and dissociation phases (black line) of data shown in (A), together with fits to a bivalent analyte model (red line), with k_a = 2.25 × 10^6 M^{-1} s^{-1} and k_d = 57.93 × 10^{-3} s^{-1}. Analysis temperature was 25 °C.

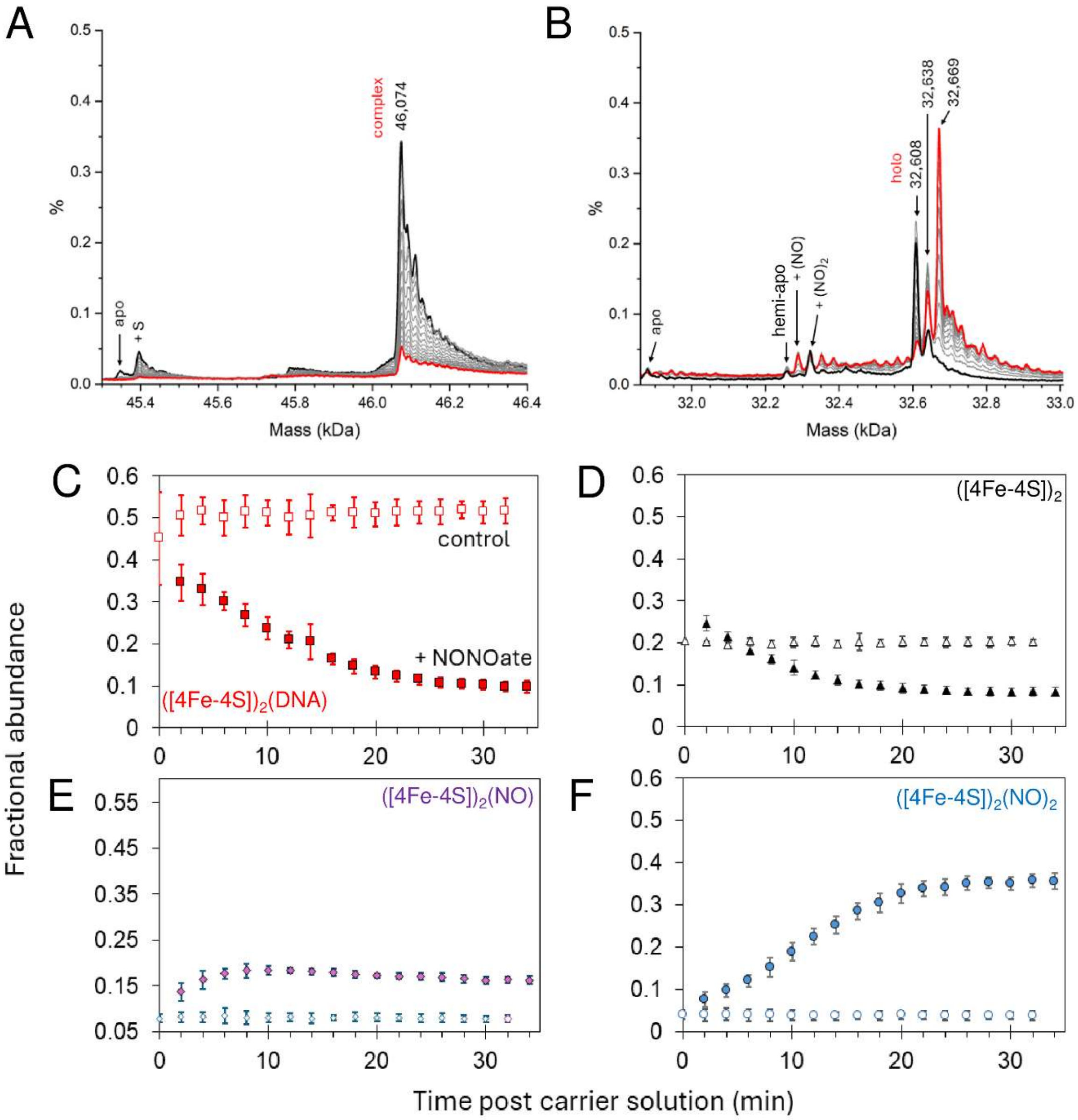

Figure S7. Wide range deconvolution of in situ nitrosylation spectra. Regions corresponding to **A)** *NsrR-hmpA1* complexes and **B)** NsrR dimers, before (black like) and after (red line) the addition of ~3.5 [NO]:[4Fe-4S] cluster, as DEA-NONOate. Intervening spectra (grey) correspond to the incremental additions of ~0.2 NO per cluster. The data are from the same experiments as those of Fig. 7, and are 2 min averages of collected data. **C - F)** Comparison of fractional abundance for temporal behaviour of *NsrR-hmpA1* samples treated with carrier solution in the presence (filled symbols) and absence (open symbols) of DEA NONOate. Sufficient DEA NONOate was added to release ~0.2 [NO]:[4Fe-4S] over each 2 min period.

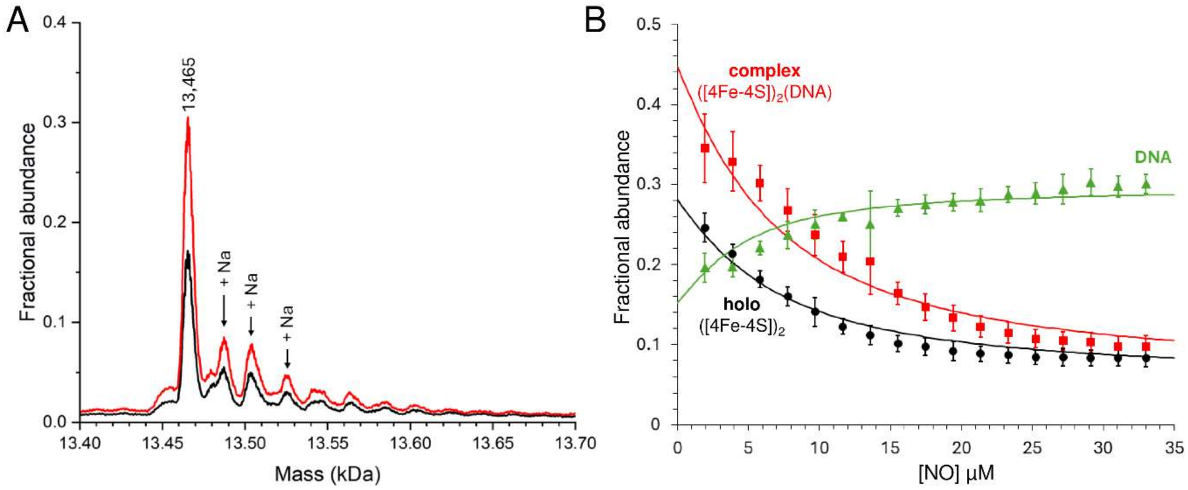

Figure S8. Nitrosylation of NsrR-*hmpA1* complexes probed by native MS. A) Deconvoluted native MS spectral region corresponding to unbound DNA before (black like) and after (red line) the addition of ~3.5 [NO]:[4Fe-4S] cluster, as DEA-NONOate. Intervening spectra due to the incremental additions of ~0.2 NO per cluster are omitted for clarity. The data are from the same experiments as those of Fig. 7, and are 2 min averages of collected data. **B)** Average (n=4) fractional abundance of unbound DNA as a function of the [NO][4Fe-4S] ratio. Error bars represent standard deviations. Data from Figure 7 for the NsrR-*hmpA1* complex (red squares) and holo NsrR dimers (black circles) are shown for completeness. Solid lines represent fits using a simple equilibrium model (see Methods). Samples contained 8 µM [4Fe-4S], 8 µM *hmpA1* DNA.

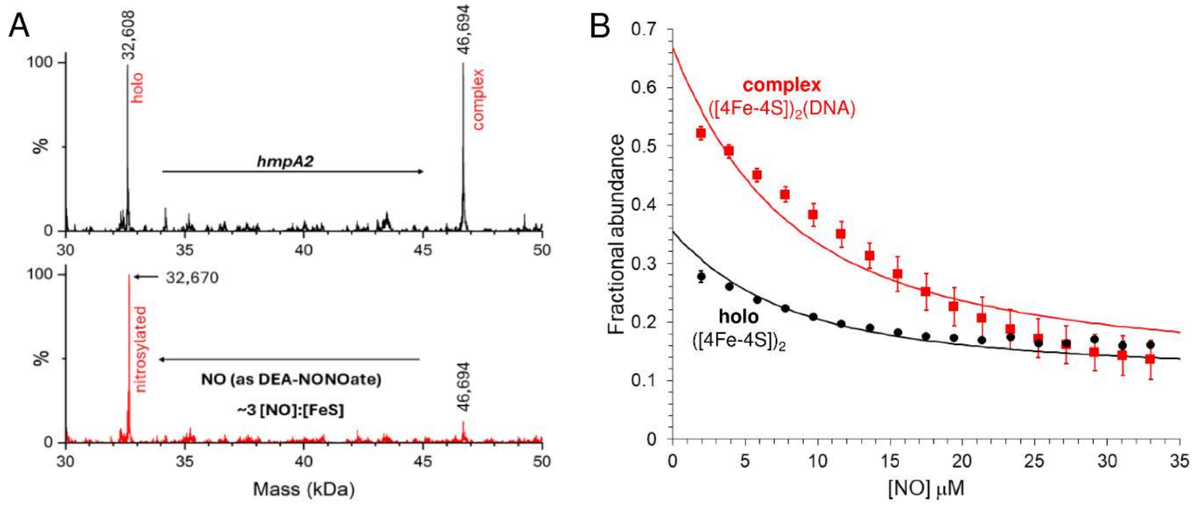

Figure S9. Nitrosylation of NsrR-*hmpA2* complexes probed by native MS. A) Comparison of NsrR-*hmpA2* complexes before (black line) and after (red line) the addition of NO (as DEA NONOate). The pre-addition spectrum contains both bound and unbound NsrR, consistent with relatively low affinity of NsrR for *hmpA*2. **B)** Average (n=2) fractional abundance for the NsrR-*hmpA2* complex (red squares) and holo NsrR dimers (black circles) as a function of the [NO][4Fe-4S] ratio. Error bars represent standard deviations. Solid lines represent fits to the same equilibrium model used for *hmpA1*. Samples contained 8 μM [4Fe-4S], 8 μM *hmpA2* DNA.

Supporting references

1. K. L. Kay, C. J. Hamilton and N. E. Le Brun, *Metallomics*, 2016, **8**, 709-719.
2. J. C. Crack and N. E. Le Brun, *Meth Mol Biol*, 2021, **2353**, 231-258.
3. J. C. Crack and N. E. Le Brun, *Chem Eur J*, 2019, **25**, 3675-3684.
4. A. Volbeda, E. L. Dodd, C. Darnault, J. C. Crack, O. Renoux, M. I. Hutchings, N. E. Le Brun and J. C. Fontecilla-Camps, *Nat Commun*, 2017, **8**, 15052.
